# Effective removal of textile dye via synergy of adsorption and photocatalysis over ZnS nanoparticles: Synthesis, modeling, and mechanism

**DOI:** 10.1016/j.heliyon.2024.e36949

**Published:** 2024-08-28

**Authors:** Sabri Ouni, Faiza Yahia, Naim BelHaj Mohamed, Mohamed Bouzidi, Abdullah S. Alshammari, Fahad Abdulaziz, Adrián Bonilla-Petriciolet, Mansour Mohamed, Ziaul R. Khan, Noureddine Chaaben, Mohamed Haouari

**Affiliations:** a Research Laboratory on Heteroepitaxy and Applications, University of Monastir, Monastir, Tunisia; b Chongqing Key Laboratory of Multi-scale Manufacturing Technology, Institute of Green and Intelligent Technology, Chinese Academy of Sciences, Chongqing, People's Republic of China; c Chongqing School, University of Chinese Academy of Sciences, Chongqing, 400714, People's Republic of China; d University of Chinese Academy of Sciences, Beijing, 100049, People's Republic of China; e Department of Physics, College of Science, University of Ha'il, Ha'il, P.O. Box 2440, Saudi Arabia; f Department of Chemistry, College of Science, University of Ha'il, Ha'il, 81451, Saudi Arabia; g Instituto Tecnológico de Aguascalientes, Aguascalientes, 20256, Mexico; h Department of Physics, Faculty of Science, Assiut University, Assiut, 71516, Egypt; i Laboratory of Advanced Materials and Interfaces, University of Monastir, Monastir, Tunisia

**Keywords:** Nanocrystal, Photocatalysis, Statistical physics modeling, Adsorption, Methylene, Blue, Water remediation

## Abstract

In this work, we prepared sulfur-zinc nanoparticles (ZnS-TGA) functionalized with thioglycolic acid by a hydrothermal method and tested their photodegradation ability by solar irradiation. ZnS-TGA were characterized by Fourier transform infrared spectroscopy (FTIR), X-ray diffraction (XRD), high-resolution transmission electron microscope (HR-TEM), UV–Vis spectrophotometer and photoluminescence spectroscopy. In the characterization of these nanoparticles, thioglycolic acid proved to be a strong capping ligand, with a specific surface area of 36.82 m2/g and an average size of 7.15 nm. To test the photocatalytic degradability of the product, methylene blue (MB) was used as a model pollutant. Various operational variables were investigated, including pH, amount of nanoparticles, dye concentration, contact time and temperature. The equilibrium adsorption tests, and the statistical physical calculations allowed the analysis of the energetic and steric variables of the adsorption of MB dye molecules on the surface of these nanoparticles. The equilibrium data were well fitted with Langmuir-Freundlich (L-F) and the adsorption kinetics with pseudo-first order. The maximum adsorption capacity of the MB dye removal process was 30.92 mg g−1 at pH 7 and 298 K, and this process was spontaneous and exothermic. The dye molecules and the surface of the nanoparticles exhibited physical interactions with adsorption energies of 23.31–25.92 kJ/mol. The photocatalytic activity of these nanoparticles resulted in a dye degradation efficiency of 91.1 % in 180 min. The photocatalytic efficiency remained almost unchanged after five consecutive degradation cycles, resulting in a methylene blue degradation of 85 %. According to these results, these environmentally friendly nanoparticles have the potential to purify industrial and urban liquids contaminated with harmful organic compounds such as dye molecules.

## 1 Introduction

Nanotechnology, also known as nanoscience, is based on the study of the preparation routes, application, and property characterization of different materials of nanometric dimensions. It is of great interest in several application fields, including electronics [1], photocatalysis [2], biology [3] and medicine [4], and is considered to be the origin of a fourth industrial revolution. During the last twenty years, the quantum dots (QDs), which are fluorescent semiconductor nanoparticles, have been classified as promising materials because of their interesting electronic, optical and structural properties, which depend on their size and quantum confinement, size and surface-to-volume ratio (Li and He 2021). These properties have made nanoparticles (NPs) as a new class of fluorescent nanoprobes that differ profoundly, in terms of performance, from the corresponding solid materials and conventional organic dyeing materials. NPs are characterized by narrow emission spectra, continuous absorption band, chemical stability, and high photobleaching resistance. In addition to their interesting fundamental properties, these NPs have can have different technological applications [5]. They are likely to give rise to a new generation of electronic [6], optoelectronic [7] and biological devices [8], with a major impact on diverse sectors including lighting [9], sensors [10], lasers [11], photovoltaics [12], biomarkers [13] and medical imaging [14]. An intensive research has performed on the study and development of group II-VI binary NPs, in particular, those containing cadmium (e.g., CdSe, CdS, CdTe) [[15], [16], [17]]. These NPs are characterized by a fluorescence emission that can be modulated by controlling the width of their band gap (gap energy) and their size, enabling them to cover a wide spectral range in the visible region. Despite these excellent optical properties, these NPs have limitations including the cadmium toxicity and their emission at short wavelengths (blue and UV) requires very small NPs to induce strong quantum confinement, with aim of avoiding unstable QDs with poor optical quality.

To overcome these drawbacks, the characterization and preparation of a new generation of zinc-based semiconducting colloidal nanocrystals have been studied. Zinc-based semiconducting colloidal nanocrystals are nanoscale semiconductor particles composed of zinc-based materials, such as zinc sulfide (ZnS) or zinc selenide (ZnSe) [18,19]. The quantum confinement effect generates that they can display relevant electronic and optical properties. The small size of nanocrystals (NCs) causes the corresponding quantum confinement effect of the holes and electrons within a limited volume. As a result, the energy levels of the confined charge carriers become discrete, and the bandgap of these materials can be tuned by controlling the NCs size [20]. This tunability allows the precise control of the emission wavelengths and absorption of the QDs, favoring their utilization in several fields. Zinc-based QDs have been extensively studied for their optical properties, particularly their photoluminescence (PL). When they are excited with light, QDs can emit light at specific wavelengths determined by their size, which can range from ultraviolet (UV) to near-infrared (NIR) depending on the material composition [21]. This property allows a wide spectrum of applications of these materials, including optoelectronic devices, and biological imaging. Among the various zinc-based semiconductors. Indeed, these NCs are synthesized using various methods depending on the desired material and properties including chemical precipitation [22], solvothermal/hydrothermal [23], thermal decomposition [24], chemical vapor deposition [25] and electrochemical synthesis [26]. Note that the synthesis route to be applied relies on the desired NCs material, size, shape, and properties, as well as the specific application requirements. When the size of a bulk semiconductor is reduced, its surface may show imperfections due to the presence of sub-coordinated atoms on the surface, which can significantly affect the NCs optical performance. The presence of new energy levels in the energy diagram are associated with these defects. They can lie between the conduction and valence bands. They trap charge carriers, giving rise to radiative and non-radiative recombination that reduces the quantum yield (QY). Most bare semiconductor NCs displayed QY < 10 % due to a large amount of surface defects [27]. To ensure the passivation of these dangling bonds on the surface, organic ligands or inorganic shells must be used to obtain fluorescent NCs. Ligands can reduce the number of defects (sub-coordination) and passivate the surface [28]. The most common ligands used for the NCs stabilization are layers of organic thiol ligands. They include L-cysteine (L-cyst) [29] and mercaptosuccinic acid (MSA) [30]. These compounds are adsorbed on the material surface generating the passivation of surface defects and colloidal stability. They can be capped using plant extract [31], chitosan [32], Schiff base [33], and polyvinyl alcohol (PVA) [34]. The use of ligands is one of the most widely employed strategies to control the particle size distribution and shape, ensuring colloidal stability in solution and preventing nanoparticle aggregation. Note that a good control of NCs growth is ensured by the exchange of ligands on the particle surface. The functionalization with ligands improves the dispersibility of ZnS NPs in aqueous solutions, minimizing aggregation and conserving a broad surface area for photocatalysis, resulting in increased efficiency under natural sunlight exposure.

On the other hand, the water pollution generated by improper handling of the textile industry wastes is a real problem worldwide, with different consequences and implications depending on a country's socioeconomical context. The various conventional processes used to decontaminate wastewater, whether physical [35], chemical and/or biological [5,36], show an important drawback because they transfer the pollutant(s) from the fluid to a new phase, by forming a concentrated residual sludge that also generates a waste disposal problem, or the need to regenerate the materials applied in the separation, which is often costly. The most recent advances in water purification have focused on the oxidation of organic chemicals that are contained in waste effluents from textile industrial sector. In particular, the advanced oxidation processes generate biologically degradable compounds or the complete mineralization of organic molecules to obtain CO_2_ and H_2_O via the formation of chemical species that are highly reactive and effective to degrade the target pollutants [37]. The formation of HO hydroxyl radicals is a key parameter in these processes, which have a greater oxidizing performance than traditional oxidants. These radicals are capable of partially or fully mineralizing most organic compounds. Photocatalysis is a chemical process that utilizes light energy to facilitate a reaction, typically involving a catalyst known as a photocatalyst. The photocatalyst absorbs light creating electron-hole pairs that participate in the chemical reactions. The most used photocatalysts are semiconductors, particularly TiO_2_, CuO, Fe_3_O_4_ and ZnS [[38], [39], [40], [41], [42]]. It can be applied in organic synthesis, energy generation, self-cleaning surfaces, air pollution control, and water purification [43,44]. For example, the photocatalysis can be used in water purification to degrade organic pollutants such as dyes via oxidation reactions initiated by the material used as photocatalyst [45]. ZnS NPs can destroy a wide spectrum of organic pollutants, including colors, making them flexible for diverse environmental remediation applications.

In the present study, the authors have prepared environmentally friendly colloidal ZnS NPs with thioglycolic acid (TGA) and hydrothermal route. These NPs were used in photodegradation and adsorption of methylene blue (MB) as target molecule that is representative of cationic thiazine dye family. MB adsorption properties of these NPs were analyzed at different operating conditions (including temperature) and NPs were also characterized to understand their performance. Dye degradation using these NPs was evaluated under solar irradiation to assess their potential application in real systems. The goal of this study is to develop a simple and inexpensive method for synthesizing small ZnS NPs utilizing non-toxic feedstock capping agents for adsorption/photocatalytic applications. The adsorption process was analyzed via the integration of classic and statistical physics adsorption models.

## 2 Methodology

### 2.1 Chemicals

Commercial analytical grade chemicals were utilized for the experimental activities without any additional purification. Ultra-pure water (UPW, resistivity >18 MOhm), MB dye (Sigma-Aldrich, C_16_H_18_ClN_3_S ≥ 95 %, MW = 319,85 g/mol), sodium hydroxide (NaOH, 99 %), sodium sulfide (Sigma-Aldrich, Na_2_S ≥ 98 %), and zinc acetate dihydrate (Sigma-Aldrich, Zn [CH_3_COO]_2_.2H_2_O ≥ 98 %) were employed in the experiments reported in this study. UPW was produced by a Millipore System.

### 2.2 Preparation of ZnS-TGA nanocatalysts

**Fig. 1:**
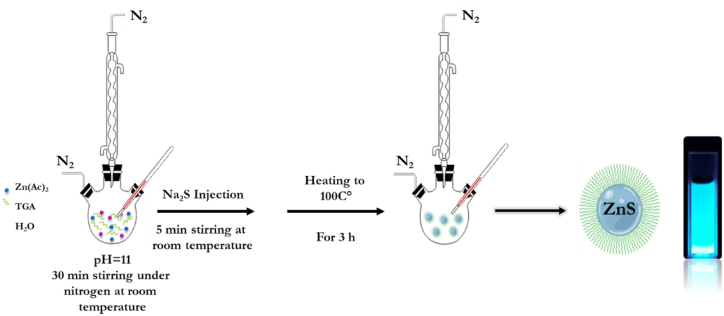
The schematic illustration of the synthesis of TGA-capped ZnS NPs.

The aqueous colloidal approach was applied to prepare TGA-capped ZnS nanocatalysts following the route given in a previous study [46]. TGA was the stabilizer to obtain the thiol-capped ZnS NPs with a Zn^2+^/S^2−^/TGA precursor ratio of 1/0.4/2.5. It should be noted that an increase in zinc concentration caused the release of several atoms of this metal generating surface interactions with the capping agent TGA. 7.5 mmol of TGA and 3 mmol of Zn[OOCCH_3_]_2_·2H_2_O were dissolved in 100 mL of distilled water using a three-necked flask. This prevented particle agglomeration and stabilized the ZnS NPs. The solution pH was adjusted to 11 with 1 M NaOH, stirred and degassed using nitrogen for 30 min. The stabilizer ligand solution and zinc acetate were stirred at room temperature and 45 mL of Na_2_S (0.8 mmol) were added. This final mixture was heated for 3 h at 100 °C, under N_2_ reflux, to obtain the ZnS-TGA NPs. A solution with a white color was produced when the reaction finished. A washing step with ethanol was applied for the final nanocolloids particles, and the suspension was centrifuged at 2500 rpm for 20 min to separate the solid phase, which was stored in a vacuum at room temperature. This NPs preparation procedure is illustrated in Fig. 1.

### 2.3 Analyses for the NPs characterization

Fourier transform infrared spectroscopy (FTIR) allowed to identify the molecules' bond vibrational frequencies associated with the presence of NPs functional groups. FTIR spectra of NPs (in their solid form) were recorded with a PerkinElmer version 5.3, covering a range of 4000–400 cm^−1^. The crystal size, d-spacing, crystal structure, and phases of NPs were characterized with X-ray diffraction (XRD) technique (Jambukiya et al., 2023). Panalytical X′ Pert Pro diffractometer was used to record the XRD powder spectrum of the NPs sample using Cu Kα (λ = 1.542 Å) emission. NPs morphology was observed with High-Resolution Transmission Electron Microscope (HR-TEM). The aqueous solution was placed on a copper grid supported by carbon film to analyze the sample. HR-TEM images were recorded with a Panalytical X′ Pert Pro diffractometer and Energy Dispersive X-ray (EDX) system. This system was operated at an accelerating voltage of 200 Kv for analyzing the elements present in NPs. UV–visible absorption spectra of NPs were obtained using SPECORD 210 Plus spectrophotometer with a quartz cuvette in the 200–800 nm wavelength range. A 325 nm helium-cadmium laser was used in the photoluminescence experiments at room temperature.

### 2.4 Dye adsorption experiments

MB is a synthetic cationic dye with a deep blue color. It has been used extensively in various scientific, medical, and industrial applications [46]. Therefore, MB was selected as pollutant model molecule to represent the pollution caused by industrial dyes. Different conditions of temperature (298–318 K), adsorbent dosage (0.1–2 g/L), contact time (0–120 min), initial dye concentration (10–30 mg/L), and MB solution pH (5–9) were assessed to quantify their effect on the adsorption process. The content of MB in the aqueous solutions was quantified using UV–vis spectroscopy with a SPECORD 210 Plus spectrophotometer operated at *λ*_max_ = 660 nm. The adsorbed MB amount (Q_e_, mg/g) was calculated with Eq. (1) [21]:

(1) $$Q_{e}=\frac{\left(C_{0}-C_{e}\right)\;V}{m}$$

(2) $$Dye\;Adsorption\;efficiency\;\left(\%\right)=\frac{\left(C_{0\;}-C_{e}\right)}{C_{0\;}}x\;100$$

The MB equilibrium and initial concentrations (mg/L) are given by Ce and C_0_, m represents the NPs mass (mg), and V is the volume of dye aqueous solution (mL). The plot of Q_**e**_ versus C_e_ corresponds to the isotherm representing the adsorption equilibrium for tested system.

On the other hand, the X-ray density *ρ* (g/cm^3^) of hexagonal or cubic NPs was obtained with Eq. (3) [19]:

(3) $$\rho =\frac{ZM}{N_{A}V_{cell}}$$

where *N*_*A*_ is the Avogadro number, *V*_*cell*_ is the unit cell volume (cm^3^), *Z* is the number of atoms per unit cell and *M* (g/mol) is the ZnS molecular weight. Equation (4) was utilized to calculate the specific surface area *S* (m^2^/g) of ZnS NPs [19]:

(4) $$S=\frac{6}{\rho D}$$

where *D* (nm) is the average size of NPs.

### 2.5 Sunlight-based photocatalytic degradation experiments

A photochemical reactor was used in the dye degradation experiments under sunlight irradiation. Suspensions of the 30 mg of ZnS photocatalysts and 30 mL of MB dye solution (with different concentrations) were used for these photocatalytic experiments. They were performed at pH 7 and 300 K. After the adsorption phase (in the dark), the dye degradation was evaluated via the sunlight exposition (between 11 a.m. and 2 p.m.) of these suspensions. For midday on a clear day in Tunisia, the intensity of sunlight at the Earth's surface is typically around 1000 W per square meter (W/m^2^). It is convenient to note that, under ideal sunlight conditions, UV radiation only makes up 5 % (50 W/m^2^) of the total solar flux received at the earth surface [19].

Photocatalytic activity was tested by monitoring the dye concentration in 2 mL samples obtained at different periods of solar irradiation (0, 10, 20, 30, 45, 60, 120, 180 min). The efficiency, the degradation rate and the half time for degradation were determined with equations (5), (6), (7):

(5) $$\eta =\frac{C_{0}-C_{t}}{C_{0}}\;x\;100$$

(6) $$kt=\mathrm{Ln}\;\left(\frac{C_{0}}{C_{t}}\right)$$

(7) $$t_{1/2}=\frac{\mathrm{Ln}\left(2\right)}{K}$$

where *k* (min^−1^) is the apparent reaction rate constant and $C_{t}$ is the final dye concentration (mg/L) after sunlight irradiation for a given time *t* (min). A linear data fitting based on *Ln* (*A/A*_*0*_) or *Ln* (C_t_/C_0_) versus *t* was used to calculated *k* where ${A\;and\;A}_{0}$ are the absorbances of MB dye solution after and before irradiation.

## 3 Results and discussion

### 3.1 NPs surface characterization

**Fig. 2:**
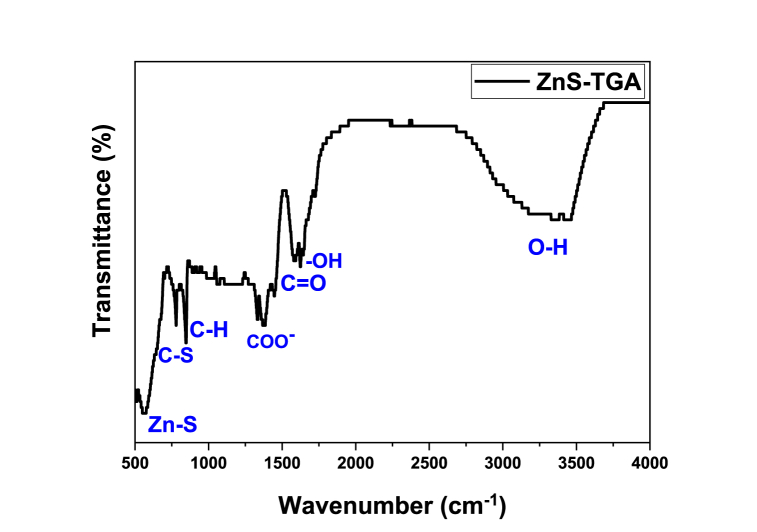
FTIR spectrum of TGA -capped ZnS NPs.

Fig. 2 displays the FTIR spectra where their results allowed to identify the stabilizing process utilizing thioglycolic acid molecules. The absorption band range of 2550–2670 cm^−1^ indicated the presence of SH bonds in TGA ligands [47]. The breaking of this bond and the corresponding loading of thiol molecules on NPs surface were associated with the changes observed for this band in the nanoparticle spectra. Zn–S stretching vibrations were identified with the absorption band at 563 cm^−1^ [20]. The sulfonate groups on TGA-capped ZnS NPs was verified by the absorption bands at 780 cm^−1^ (–C–S), 850 cm^−1^ (–S-H), 1429 cm^−1^ (–COO-), and 1508 cm^−1^ (– C=O) [47]. The O–H elongation vibration of TGA molecules was identified via the wide absorption band at 3340 cm^−1^ [19]. These findings showed that the TGA ligand was attached to the ZnS surface.

**Fig. 3:**
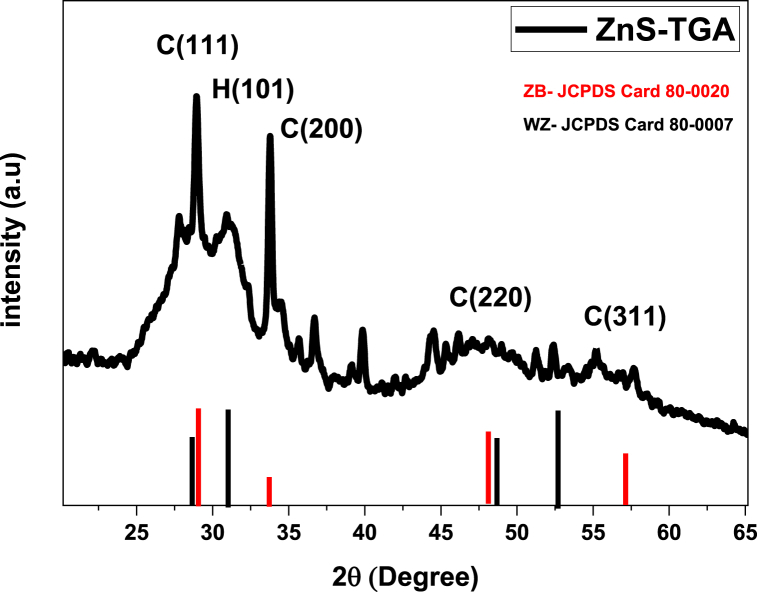
DRX pattern of TGA-capped ZnS nanocrystals.

XRD pattern of the ZnS-TGA NPs is shown in Fig. 3. The large diffraction peaks demonstrated the NPs nanometric size. The diffraction standards of wurtzite (JCPDS card No. 80-0020) and zinc blende (JCPDS card No. 80-0007) phases were used for comparison and analysis of NPs results. The diffraction peaks located at 2θ = 28.95°, 33.76°, 47.58°, and 55.27° were associated with the diffraction planes (111), (200), (220), and (311), respectively, of the zinc blende phase. The (101) crystallographic wurtzite ZnS plane was identified via the weak peak at 31.08°. It was determined that even though the hexagonal phase's contribution to the XRD pattern was minimal, its existence cannot be ruled out. Therefore, symmetry circumstances favoring the most preferred (111) direction led to the nucleation of cubic crystal structure rather than hexagonal structure [46]. To provide a more precise measurement of the stabilizer's impact, the average size D and full width at half maximum (FWHM) of TGA-capped ZnS NPs were estimated by fitting the XRD pattern to a theoretical profile. The Debye-Scherrer (Eq. 8.) fitted the Gaussian profile to the Bragg peaks obtaining FWHM. This parameter allowed to calculate the average crystallite size (D, nm) [48]:

(8) $$D=\frac{K\lambda }{\beta \;\cos\;\left(\theta \right)}$$

where β is the complete width at half maximum of the diffraction peak in radians, K is the shape factor (0.9), and θ is the Bragg diffraction angle. Note that the X-ray wavelength was 1.54 Å. The computed average crystallite size was 7.15 ± 0.1 nm. The lattice constants of the hexagonal and cubic phases were obtained from Equations (9), (10) [49]:

(9) $$d_{hkl}^{2}=\frac{a_{c}^{2}}{h^{2}+k^{2}+l^{2}}$$

(10) $$d_{hkl}^{2}=\frac{1}{\frac{4\left(\;h^{2}+hK+K^{2}\right)}{3a_{h}^{2}}+\frac{l}{c_{h}^{2}}}$$

where (a_c_, a_h_, and c_h_) are the lattice constants of the cubic and hexagonal phases of nanocrystals, (hkl) are the Miller indices, and d_hkl_ is the inter-reticular distance that is provided for both structures. The lattice parameters with the average calculated values were: a = 3.8°A, c = 6.9°A (hexagonal), and a = 5.4°A (cubic). The Scherrer formula provided a restricted value of the NCs size and accounted for the size effects related to the diffraction data. Bhattacharjee and Chattopadhyay (2002) have indicated that this model ignores other parameters, such as lattice strain, dislocation density, and stacking fault that could be used to rectify NC sizes [50]. Therefore, Equation (11) calculated these variables, and Table 1 lists the estimated values [19].

(11) $$\varepsilon =\frac{\beta \;\cos\;\left(\theta \right)}{4}\;;\delta =\frac{1}{D^{2}}\;;\;SF=\frac{{2\pi }^{2}}{{45\left(\tan\;\theta \right)}^{\frac{1}{2}}}\beta_{hkl}$$

**Table 1:** Structural properties of TGA-capped ZnS nanocrystals.

Sample	Crystallite size D (nm)	Dominant Plans d_hkl_	Lattice constant (Å)	Structure	Strain (*ε*)	Dislocation density (δ) (lines/m^2^)x10^15^	Stacking fault (SF)
**ZnS-TGA**	7.15	C (111)	a = 5.4	ZB	0.00698	19.56	0.014
			a = 3.8	WZ			
			c = 6.9				

The term lattice strain (*ε*) describes the regularity that is distorted or altered due to crystal flaws like lattice [51]. Therefore, the amount of flaws in the nanocrystal is explained by the SF and δ [52]. The value of dislocation density indicated that the semi-conductor nanocrystals were less ordered due to small size. Therefore, the highest possibility of dislocations was due to small NPs size since they tend to stabilize their higher surface energy. The structural strain is linked with the NCs surface stress from the TGA capped surface during the relaxation and growth of atomic positions at SF interface [30].

**Fig. 4:**
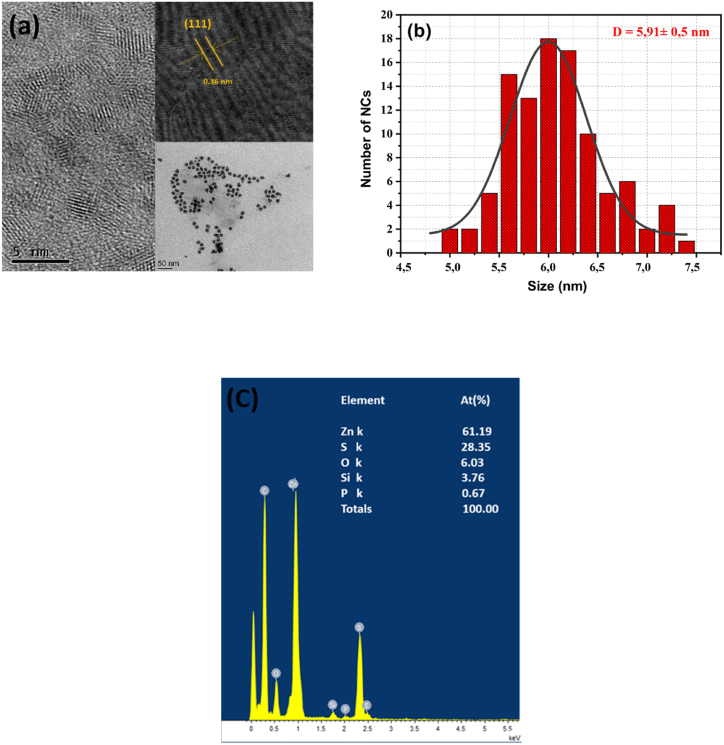
(a) HR-TEM images of TGA-capped ZnS NPs with an inset showing the 0.36 nm lattice spacing that corresponded to the (111) plane, (b) particle size distribution measured from 100 QDs, and (c) EDX results.

Fig. 4(a) reports the HR-TEM measurements to analyze the particle size and morphology of ZnS-TGA NCs. These nanocrystals displayed a spherical shape and the estimated average ZnS-TGA diameter was 5.91 ± 0.5 nm (Fig. 4(b)**)**. The interplanar distance measured for NCs was 0.36 nm and this value was close to the (111) plane of zinc blende ZnS (0.312 nm). Fig. 4(c) displays the elemental nanocrystals composition where S and Zn were the major elemental components.

### 3.2 Optical characterization of TGA-capped ZnS NPs

**Fig. 5:**
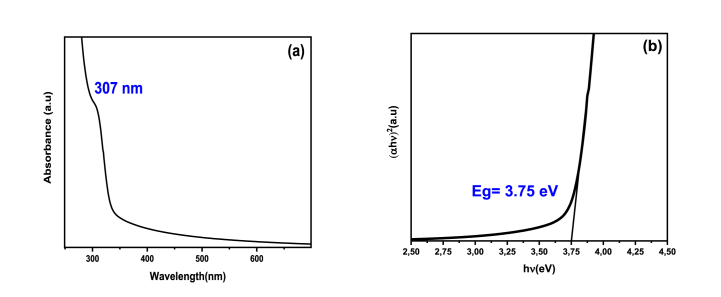
(a) Optical absorption spectrum and (b) graphical method for calculating the optical gap of TGA-capped ZnS NPs.

Fig. 5(a) shows the absorption spectrum of NPs. This spectrum contained broad absorption bands in the UV region that extended to the visible region. The first electronic transition 1Se-1S_h_ was associated to the absorption edges at 307 nm [53]. This spectrum showed a blue shift due to the size effect of the ZnS-TGA NPs, which differed from the response obtained for the bulk ZnS (344 nm). Eq. (12) calculated the NPs band gap energy (*Eg*) [54]:

(12) *αhν* = A (hν-Eg)^n^

where A is a constant, α is the absorption coefficient, and *hυ* is the incident photon energy. The parameter n is a function of the transition type, which is equal to ½ for direct semiconductors. The extrapolation of the tangent of the near edge band allowed the determination of the band gap energy, see Fig. 5(b). The band gap was 3.75 eV with a blue shift due to the quantum confinement effect. This optical behavior was different than that obtained for bulk ZnS (Eg = 3.6 eV). ZnS-TGA NCs showed λ > 300 nm and this result suggested that they can be used in solar-irradiation-based applications.

**Fig. 6:**
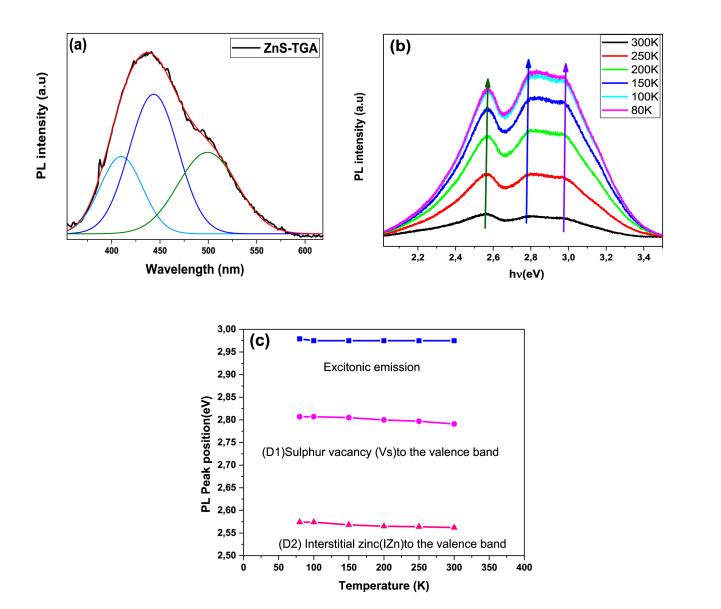
(a) Gaussian adjustment of PL spectra, (b) PL spectrum as a function of temperature and (c) PL peak position as a function of temperature for both *D*_*1*_ and *D*_*2*_ defects for ZnS-TGA NPs.

The emission spectrum of TGA-capped ZnS QDs colloidal aqueous solution after helium-cadmium laser irradiation (325 nm) at 20 °C is reported in Fig. 6(a). At around 440 nm, the photoluminescence spectrum exhibited a broad and strong band with an FWHM of approximately 77 nm. The small NPs size and the large surface-to-volume ratio of the imperfect surface passivation resulted in a high size distribution where both were the causes of the high FWHM value. The primary cause of either extensive (aggregates, cavities, dislocations, etc.) or punctual (interstitial, substitutional, vacancies, etc.) defects was the synthetic precursor stoichiometric ratio and the development process [53]. A Gaussian function was applied to deconvolute ZnS-TGA QD emission spectrum by considering three primary bands in the visible range: 495 nm (2.50 eV), 440 nm (2.82 eV), and 408 nm (3.04 eV). The band at 408 nm was associated to the direct band-to-band recombination, which was related to blue emission. It was proposed that the blue luminescence was caused by electrons localized on sulfur vacancies (V_s_) with holes in the valence band transitioning into the band at 440 nm [20]. The contribution from cadmium interstitial of (*I*_*Zn*_) to the valence band (VB) was ascribed to the 495 nm band of green emission [46]. The significant emission was attributed to the zinc interstitial and sulfur vacancy generated in the ZnS-TGA NPs, which played an important role in the photodegradation process. Note that the reaction temperature, capping agent concentration, refluxing duration, and capping agent concentration affect the photoluminescence performance. Fig. 6(b) reports the PL spectra of NPs in the aqueous solution that were recorded at 80–300 K. Fig. 6(c) displays the PL peak position (in eV) versus temperature. The emission spectra of ZnS QDs showed a broad band at 416 nm (2.98 eV) (band edge "BE") besides two low-energy broad bands at 2.81 eV (441 nm) (defect 1) and 2.56 eV (484 nm) (defect 2) at tested temperatures. PL spectra of QDs obtained at low- and room-temperatures were similar. The ZnS-TGA QDs excitonic state induced considerable blue shifts as the temperature decreased and, consequently, the PL bands were narrower while increasing in their intensity. The emission of defects and FWHM of band edge increased with temperature but with a minor shift in their maximum location. Varshni model [55] was used to calculate the weak blue shift of PL peak location. The result was 0.4 meV as the temperature decreased from 300 to 80 K. This trend has been observed for different semiconductors in the same temperature range and represented the energy band gap's shrinkage caused by the increment of temperature because of the lattice's thermal expansion and exciton-phonon interaction [56]. The band gap was widened by these processes, which in turn caused the excitonic emission to shift blue. However, the energy positions of D1 and D2 bands were slightly altered due to the temperature increment. This tendency was most likely caused by the QDs size distribution and the strong attachment of impurity levels (surface defects or trap state) to the ZnS-TGA lattice near the forbidden band gap [57]. Conversely, the intensities of excitonic emission and trapping increased, particularly at low temperatures. The suppression of phonon-coupled thermal quenching was associated with this result and the different temperature sensitivity of the excitonic and trapping states in QDs [58]. The phonon coupling strength also increased with temperature. Consequently, the non-radiative recombination probability of holes and electrons was linked to the phonon absorption and PL intensity reduction [59].

### 3.3 Impact of operating conditions on MB dye adsorption on NPs

**Fig. 7:**
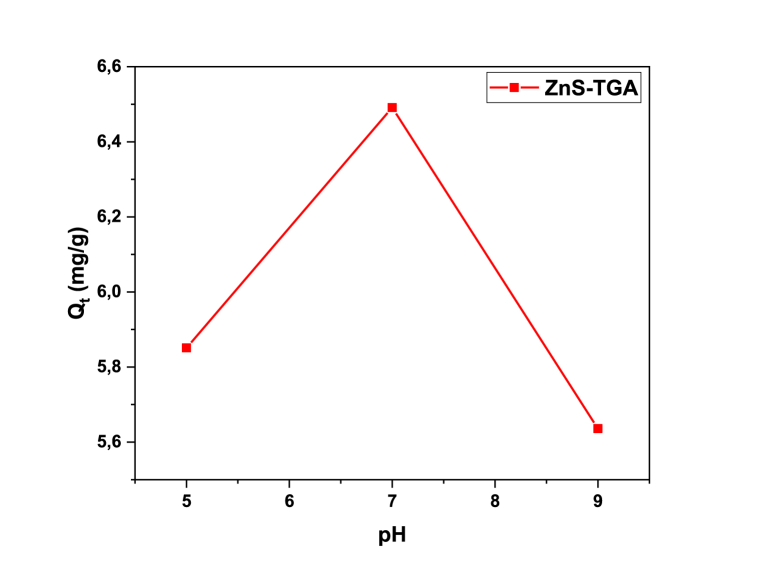
Effect of solution pH on the MB adsorption using TGA-capped ZnS NPs.

**Fig. 8:**
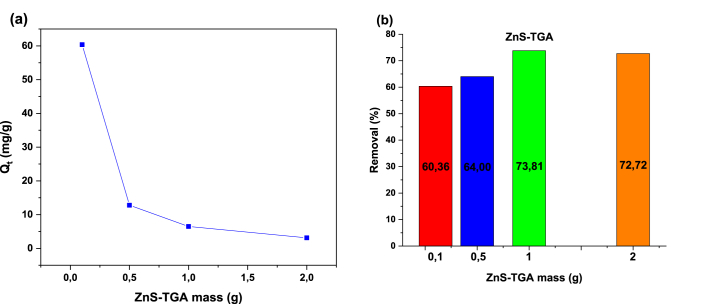
(a**)** Effect of ZnS-TGA mass on the MB adsorption capacity and (**b)** removal efficiency.

Fig. 7 reports the dependence of MB adsorption with respect to the aqueous solution pH. These studies were performed with 10 mg/L dye concentration, 1 g/L of NPs dosage and pH 5–9. Dye removal (Eq. (2)) increased from 58.51 to 64.91 % when solution pH changed from 5 to 7. This increment on the dye removal was caused by electrostatic interactions between the negatively charged NPs and the positively charged MB molecules [19]. In contrast, the dye removal decreased with further increments until pH 9. Consequently, the best adsorption condition for MB dye was pH 7 (see Fig. 8).

Fig. 8(a) and (b) reports the impact of NPs mass on MB adsorption. The mass of the nano-adsorbents varied from 0.1 to 2 g, while the other operating conditions were fixed, i.e.: 298 K, pH 7, 10 mg/L dye concentration, and 120 min contact time. The dye removal improved with the increment of NPs mass because the number of adsorption sites available increased with the specific surface area, thus favoring dye adsorption [60]. It was determined that MB removal rate was 74 % when using 1 g of organic TGA-capped ZnS NPs. This may be explained by a greater number of adsorption sites as well as better NP dispersion in the aqueous solution. The adsorption capacity of NPs decreased by a further addition of adsorbent mass. For high NPs dosages, the active sites with higher energy become less available, resulting in the occupation of low energy active sites and the reduction of adsorption capacity [61]. Hence, 1 g/L of NPs was selected for the dye removal studies.

**Fig. 9:**
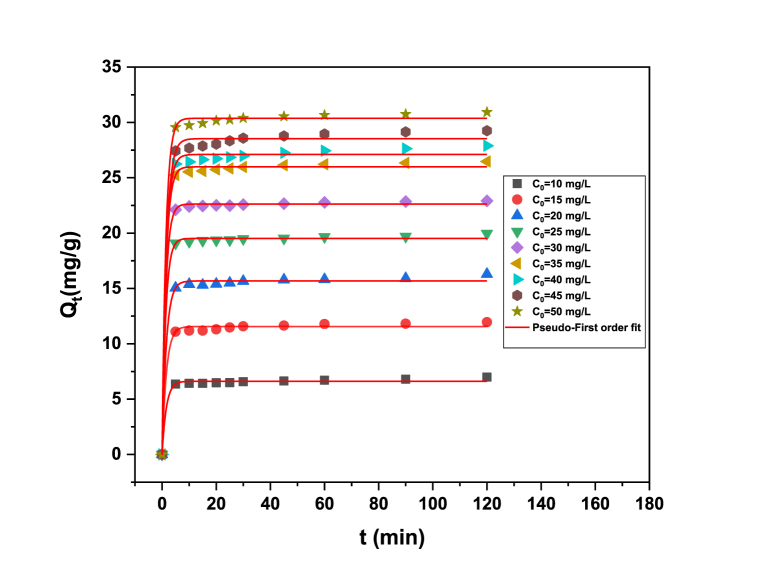
Modeling of MB adsorption kinetics using the pseudo-first order equation.

Adsorption was studied over time to determine the adsorbed dye amount at different contact periods (see Fig. 9). For the first 5 min, the removal of MB dye was fast followed by a slower dye adsorption rate from 10 to 120 min until reaching the equilibrium. This trend was associated with the high surface-to-volume ratio of the nanometer-sized adsorbents. Therefore, the maximum dye adsorption was obtained at 120 min before proceeding with the photocatalytic activity.

Fig. 9 also reports the initial dye concentration effect on the NPs adsorption performance. The increase of dye content in the aqueous solution improved the NPs adsorption capacity because the mass transfer was enhanced [62]. The results showed that the MB adsorption capacities increased from 6.99 to 30.92 mg/g when adsorbate concentration changed from 10 to 50 mg/L. This trend was associated with the high mass transfer gradient that favored the diffusion of MB molecules on NPs surface, thus enhancing the adsorption interactions.

**Fig. 10:**
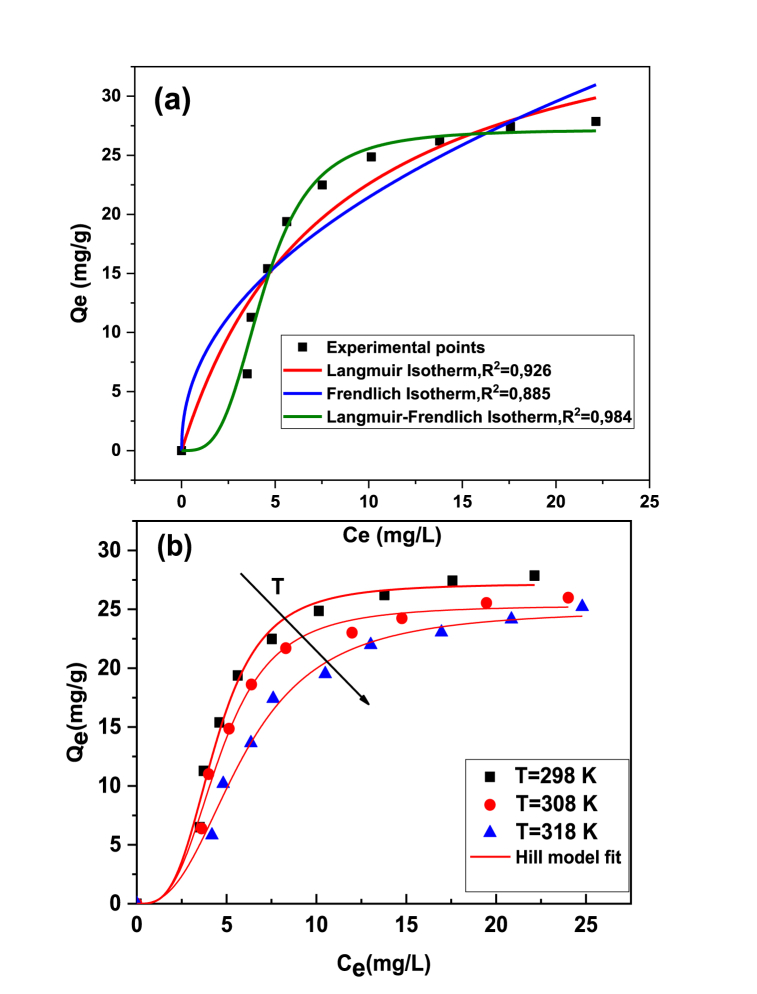
Modeling of MB adsorption isotherms of TGA-capped ZnS NPs using a) traditional isotherm equations and b) statistical physics model.

MB adsorption capacity versus solution temperature is shown in Fig. 10(b). NPs adsorption performance decreased with the temperature increment from 298 to 318 K indicating an exothermic process. This solution temperature increase reduced the binding interaction forces involved in dye removal [63]. Therefore, a solution temperature of 298 K was used as the best condition for MB dye adsorption using these NPs.

### 3.4 Dye adsorption kinetics

Dye kinetic studies using NPs and their respective modeling are shown in Fig. 9. The adsorption capacity varied from 6.61 to 30.37 mg/g in these kinetic tests when the MB concentration changed from 10 to 50 mg/L. MB dye removal was favored by NPs particle size. ZnS-TGA specific surface area was 36.82 cm^2^/g. Several kinetic models were used to calculate essential information for the application of these nano-adsorbents in the dye removal process. Two kinetic models were utilized: The pseudo-first order (PFO, Eq. (13)) and the pseudo-second order (PSO, Eq. (14)). They fitted the MB adsorption data, and the results are given in Fig. 9. PFO model is commonly applied to analyze the adsorption of water pollutants and is defined as [64,65]:

(13) *Q*_*t*_*= Q*_*e*_*(1-exp(-K*_*1*_*t))*

where K_1_ (min^−1^) is the adsorption rate constant, Q_e_ (mg/g) is the calculated equilibrium adsorption capacity (mg/g) and Q_t_ (mg/g) is the experimental adsorption capacity quantified at time t. PSO model is a type of kinetic model that describes the rate at which an adsorption process occurs, which is given by the next expression [61,66]:

(14) $$Q_{t}=\frac{Q_{e\;}^{2}K_{2}t}{1+Q_{e}K_{2}t}$$

where *K*_2_ (mg/g⋅min) is the corresponding PSO rate adsorption constant. Table 2 provides the kinetic modeling results. The best equation for the correlation of adsorption kinetic data was chosen using the R^2^ value. The modeling results indicated that PFO was the best kinetic to fit the MB dye adsorption on NPs with R^2^ = 0.99. Calculated Qe values using this model were closer to the experimental ones. Note that the dye adsorption kinetic implied two stages: dye diffusion on NPs surface and the adsorbent saturation at equilibrium condition.

**Table 2:** Pseudo-first-order kinetic parameters for the MB adsorption on TGA-capped ZnS NPs.

Model parameter	Initial dye concentration (mg/L)								
	10	15	20	25	30	35	40	45	50
K_L_ (min^−1^)	0.64	0.65	0.70	0.75	0.78	0.81	0.84	0.88	0.92
Qe (mg/g)	6.62	11.55	15.65	19.52	22.63	25.99	27.10	28.53	30.37

### 3.5 Dye adsorption isotherms

Fig. 10(a) reports the experimental MB adsorption isotherm and the results obtained from the corresponding data correlation. Langmuir-Freundlich, Langmuir and Freundlich equations were used to fit the dye adsorption equilibrium. A monolayer adsorption process is assumed by the Langmuir isotherm where the adsorption occurs on a limited number of identical adsorption sites. It is mathematically using the equation (15) [67]:

(15) $$Q_{e}=Q_{\max}\frac{K_{L}C_{e}}{1+K_{L}C_{e}}$$

where *K*_*L*_ (L/mg) is the Langmuir adsorption energy and *Q*_*max*_ (mg/g) is the NPs monolayer adsorption capacity. The empirical Freundlich model accounts for heterogeneous surfaces and usually describes a multilayer adsorption with equation (16) [68]:

(16) $$Q_{e}=K_{L}C_{e}^{n_{F}}$$

where *n*_*F*_ is the adsorption intensity parameter, and *K*_*F*_ (mg/g) is the Freundlich adsorption constant. The Langmuir -Freundlich (L-F) isotherm equation is based on the previous isotherms that can describe heterogeneous surfaces. As shown in equation (17), this isotherm can be expressed as follows [21]:

(17) $$Q_{e}=\frac{Q_{\max}{\left(K_{LF}C_{e}\right)}^{n_{LF}}}{1+{\left(K_{LF}C_{e}\right)}^{n_{LF}}}$$

where n_LF_ is the heterogeneity index and ${\;K}_{\text{LF}}$ is the adsorption affinity constant. The results of isotherm modelling are reported in Table 3. These isotherm models fitted the experimental dye adsorption data of capped ZnS nano-adsorbents with R^2^ = 0.88–0.98. L-F equation showed the highest R^2^ and was the best model to describe these experimental results. This model predicted Q_max_ = 33.15 mg/g, which was consistent with the experimental value of 30.92 mg/g. The results from the Langmuir model suggested that the surface of synthesized NPs samples was homogeneous for MB adsorption. Calculated n_F_ values from Freundlich model were >1 (n_F_ = 2.16) indicating that adsorption was favorable [69] and involved physical interaction forces [70,71]. These results prove that these NPs are an alternative for treatment of wastewater polluted by dye molecules.

**Table 3:** Parameters of the isotherm models for the MB adsorption on TGA-capped ZnS NPs.

Langmuir isotherm (R^2^ = 0.92)		
Q_max_ (mg/g)	K_L_	
40.68	0.12	
Freundlich isotherm (R^2^ = 0.88)		
K_f_ (mg/g)	n_F_	
7.40	2.16	
Langmuir-Freundlich (R^2^ = 0.98)		
Q_max_ (mg/g)	K_L-F_	n_LF_
33.15	0.27	3.35

On the other hand, statistical physics modeling was useful to describe the interactions between dye molecules and NPs surface, and to correlate the experimental adsorption isotherms using parameters associated with these microscopic interactions. This modelling approach was performed to improve the interpretation of MB adsorption process via the estimation of the adsorption energies, the number of adsorbed dye molecules per NPs adsorption site (*n*) and NPs adsorption site density (*D*_*M*_). A monolayer advanced model fitted the experimental isotherms of the MB dye adsorption on NPs. It assumed that the cationic dye molecules formed a monolayer on the particle surface. The layer of adsorbed MB molecules was formed due to the interactions between MB dye and NPs surface. This model (Eq. (18) also considered that the main NPs adsorption site could interact with a variable number of adsorbate molecules (superior, inferior or equal to 1) [72]. It is defined as:

(18) $$Q_{e}=\frac{n∙D_{m}}{1+{\left(\frac{C_{1/2}}{C_{e}}\right)}^{n}}$$

where C_1/2_ is the half-saturation dye concentration (mg/L). In equation (19), the adsorption energy (E^a^_1_, kJ/mol) is expressed as [73]:

(19) $$\Delta E_{1}^{a}=R.T.\ln\left(\frac{C_{s}}{C_{1/2}}\right)$$

where *Cs =* 40 mg/L is the MB dye solubility, *R* = 8.314 × 10^−3^ kJ/K⋅mol is the universal ideal gas constant and *T* (K) is the adsorption temperature.

Table 4 and Fig. 10(b) provide the results of data modelling. The calculated *D*_*M*_ values decreased with temperature from 26.45 to 10.64 mg/g. Note that if n values increased, the space in the ZnS-TGA nano-adsorbents surface reduced and, consequently, the number of adsorption sites available for dye removal became limited [19]. Indeed, a high *D*_*M*_ value implies a high effectiveness of the adsorbent since more adsorption sites are available for pollutant adsorption [21]. Calculated *n* values ranged from 1.22 to 2.40 indicating that MB dye molecules could be bound or adsorbed via a nonparallel adsorption orientation or inclined, which corresponds to a multimolecular adsorption [21]. MB dye molecules interacted with NPs surface involving an aggregation process to form trimers and dimers in the aqueous solution (i.e., *n* > 1). This multimolecular adsorption phenomenon usually takes place for dye molecules as reported in other studies [21]. The adsorption capacities at saturation (*Q*_*sat*_) decreased with the aqueous solution temperature from 32.24 mg/g at 298 K to 25.61 mg/g at 318 K. The calculated adsorption energies for this exothermic adsorption ranged from 25.92 to 23.31 kJ/mol, implying the existence of physical interactions between the ZnS-TGA nano-adsorbent surface and MB dye molecules. Hydrogen bond and electrostatic interactions could be implicated in the MB loading on NPs surface.

**Table 4:** Parameters of the Hill model for the MB adsorption on TGA-capped ZnS NPs.

T (K)	n	D_M_ (mg/g)	C_1/2_ (mg/L)	Q_sat_ (mg/L)	$E_{1}^{a}$ (kJ/mol)
298	1.22	26.45	1.14	32.24	25.92
308	1.91	13.87	2.35	26.43	24.94
318	2.40	10.64	5.91	25.61	23.31

Table 5 contains the MB adsorption capacities of other ZnS nanoparticles reported in different studies [21,[74], [75], [76], [77], [78], [79]]. These results showed that TGA-capped ZnS nano-adsorbents exhibited a higher MB adsorption capacity than those of uncovered ZnS NPs [78], ZnS encapsulated with broccoli extract [79] and ZnS encapsulated with mercaptopropionic acid [21].

**Table 5:** Comparison of MB adsorption capacities of TGA-capped ZnS NPs and other adsorbents reported in literature.

Sample	Dye	Time (min)	Q_max_ (mg/g)	Reference
ZnS-MPA	MB	120	25.18	[21]
ZnS	MB	120	15.65	[74]
ZnS-Broccoli	MB	60	9.00	[75]
Zeolite	MB	240	10.82	[76]
Cu_2_O	MB	1000	2.08	[77]
TiO2	MB	120	13.10	[78]
ZnO	MB	180	9.59	[79]
NiO	MB	180	17.10	[79]
ZnS-TGA	MB	120	30.92	This work

### 3.6 Sunlight-based photocatalytic degradation of MB dye using ZnS-TGA NPs

#### 3.6.1 Degradation pathways

**Fig. 11:**
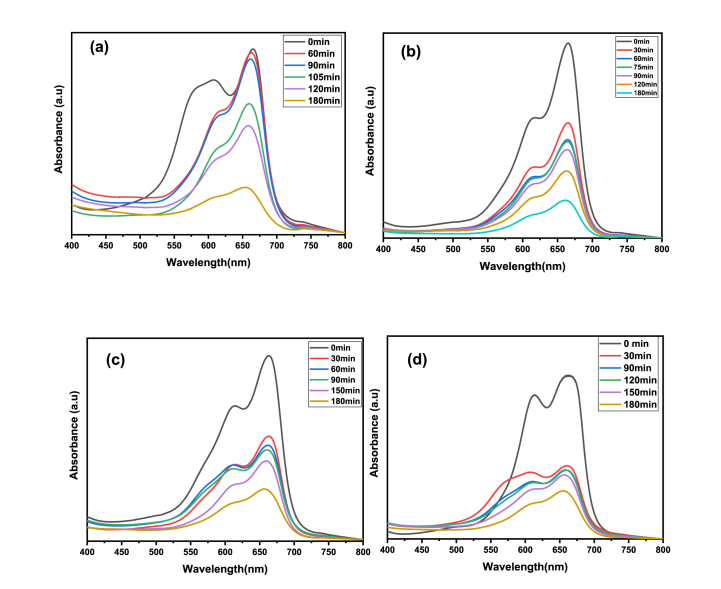
UV–visible absorption spectra of MB decolorizing using ZnS-TGA NPs under sunlight irradiation with different initials MB concentrations: (a) 10, (b) 15, (c) 20 and (d) 25 mg/L.

**Fig. 12:**
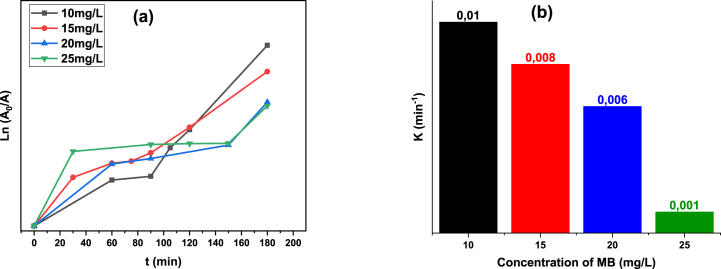
(a): Photocatalytic rate constant curves and (b) degradation rates for different MB dye concentrations.

Initial tests using the MB solution were conducted without NPs, and a marginal degradation (about 10 % in 180 min) was noted. This degradation could be caused by the dye molecules' self-sensitization light or by OH* radicals initiated from water (blank test) [80]. This finding proved that removal of this cationic organic dye by direct photolysis was ineffective and showed that dye degradation was almost negligible in the absence of photocatalysts. Fig. 11(a–d) reports the UV–vis absorption spectra of MB dye solutions containing TGA-capped ZnS NPs as a function of irradiation duration, whereas Table 6 shows dye degradation (%) obtained for pollutant concentrations of 10–25 mg/L. The degrading performance of ZnS-TGA nanocatalysts was evaluated using four different dye concentrations. The results showed that the absorbance band gradually diminished with increasing solar irradiation duration, demonstrating the disintegration of the MB chromophoric structure [81]. Consequently, the blue color disappeared due to the breakdown of the azo function (C-S^+^=C) source of the blue color and indicated that the hydroxyl radicals had attacked the aromatic compounds through the creation of radical intermediates [82]. Therefore, under solar radiation, the concentration of MB dye decreased in the presence of ZnS-TGA nanocatalysts until the initial dye solution fully discolored, signifying that MB molecules were entirely broken down the impact of the initial MB concentration on the decomposition efficacy is shown in Table 7. After 180 min under sunlight exposure, the maximum MB concentration produced the lowest photocatalytic effectiveness of 72.22 %, while the best degradation of 91.10 % was observed for 10 mg/L. The small size of TGA molecules utilized as stabilizer and the specific surface (i.e., 36.82 m^2^/g) of the nanocatalysts can be associated to these efficiencies. This high efficiency could be the result of ZnS broad band gap energy, which raised e-h pair redox potential and improved photocatalytic performance [46]. As the initial MB concentration increased, the dye degradation decreased. As the dye molecules covered the active sites, the production of OH* and O_2_*^-^ radicals on the photocatalyst surface were reduced at high dye concentrations, which was the main cause of this behavior. An additional plausible reason might be that the dye's ability to block sunlight resulted in a decrease in the nanocatalysts capacity to absorb light [46]. Dye molecules have a greater capacity to absorb sunlight than ZnS-TGA nanocatalysts, which lowered the catalytic reaction's efficiency as free radical concentrations dropped [19]. MB dye degradation rate constant was calculated using the Langmuir-Hinshelwood (L-H) kinetic model. Fig. 12(a) presents photodegradation plots of ln (A_0_/A) vs. time t for MB degradation. The lowest MB concentration (e.g., 10 mg/L) produced the best degradation constant K (i.e., 0.025 min^−1^), as shown in Fig. 12(b). The half-time value (i.e., 47.46 min) of the MB dye demonstrated an increase in photodegradation under sunlight with the use of TGA-capped ZnS nanocatalysts. The degradation efficiency decreased as the dye concentration increased; the maximum initial dye concentration resulted in a degradation rate of 0.011 min^−1^. The degradation rates were significantly influenced by the MB content in the aqueous solution. These findings verified the high photocatalytic activity of TGA-capped ZnS NPs for the removal of MB. The maximum performance values for the adsorption/photodegradation of MB utilizing other photocatalytic materials are shown in Table 8 [[83], [84], [85], [86], [87], [88], [89], [90]].

**Table 6:** MB degradation efficiency of TGA-capped ZnS NPs under sunlight irradiation using 10 mg/L of initial dye concentration.

	t (min)							
	0	10	20	30	45	60	120	180
**MB Degradation (%)**	0	21.55	28.36	41.18	43.55	59.36	67.72	91.10

**Table 7:** Calculated parameters for the sunlight irradiation-based photodegradation of MB in aqueous solution using TGA -capped ZnS NPs.

Dye concentration (mg/L)	Degradation rate constant *K* (min^−1^)	Degradation efficiency *η* (%)
10	0.011	91.10
15	0.008	84.12
20	0.006	73.95
25	0.001	72.22

**Table 8:** Comparison of MB dye degradation efficiency of various materials reported in the literature.

Sample	Irradiation	Time (min)	Degradation efficiency (%)	Reference
ZnS/RGO	Visible light	240	89.43	[83]
ZnS-PVP	Sunlight	180	49.00	[84]
TiO2/ZnO	Visible light	120	73.20	[85]
CuO/CdS	UV light	120	84.10	[86]
ZnS-A	UV light	180	93.00	[87]
Ni-ZnS	Sunlight	180	87.38	[88]
ZnS	Visible light	120	78.41	[89]
Egg-NiO	UV light	240	79.00	[90]
ZnS-TGA	Sunlight	180	91.10	This work

The sunlight-based MB degradation with TGA-capped ZnS nanocatalysts implied several stages. The first step was related to the dye molecules adsorption phase on the nanocatalyst surface. ZnS-TGA photocatalyst absorbed photons from sunlight irradiation, typically ultraviolet (UV) or visible light. An electron was advanced by this absorption from the valence band to the conduction band, resulting in the formation of an electron-hole pair. Within the ZnS photocatalyst, the absorbed energy produced an excited state in which an electron was excited to the conduction band (becoming a negatively charged electron) and a hole (a positive charge) was left behind in the valence band. High levels of reactivity were observed in the holes in the valence band and the excited electrons in the conduction band. The ZnS photocatalyst surface was the site of interactions between these charged species and molecules of adsorbed reactant. By giving their extra energy to reactant molecules such adsorbed O_2_, the excited electrons in the conduction band reduced them. This created a superoxide reactive species (O_2_*^-^), which may take part in the chemical process. Conversely, water, hydroxide ions, or other molecules on the catalyst surface may be oxidized by the holes in the valence band, producing very reactive oxygen species (such hydroxyl radicals, or OH*) that can start a variety of chemical processes. MB dye molecules can be efficiently decomposed by radical-active OH* and O_2_*^-^ species to produce intermediates such as propionic acid and malonic acid, as well as the end products CO_2_ and H_2_O. These free radicals served as potent oxidizers as well as active sites for the photocatalytic degradation of organic pollutants. By moving the electron from the conduction band back to the hole in the valence band, or closing the electron-hole pair, the photocatalyst can revert to its initial state following the degradation reaction. The photocatalyst might be utilized again because of this regeneration. Equations (20), (21), (22), (23), (24), (25), (26) summarize the primary responses that might be a part of this degradation process.

(a) Adsorption phase

(20) ZnS-TGA + MB → ZnS-TGA-MB

(b) (e^−^)-(h^+^) pair generation

(21) ZnS-TGA + hν → e_**BC**_^−^ + h_**BV**_^+^

(c) Production of superoxide and hydroxyl radicals

(22) e^−^ + O_2_→ O_2_*^-^

(23) H_2_O + h^+^→OH* + H^+^

(24) h^+^ + OH^−^ → OH*

(d) Degradation phase

(25) O_2_*^-^ + MB → Degradation products + CO_2_ + H_2_O

(26) OH*+ MB → Degradation products + CO_2_ + H_2_O

It should be highlighted that these free radicals are essential to the photocatalytic process that breaks down the MB dye. The hydroxyl radical initiated the degradation of the cationic dye molecules by attacking their C-S^+^=C bond and other bonds. According to these findings, TGA-capped ZnS NPs represent a potentially useful photocatalyst that may be made using a non-toxic feedstock and used to clean up organic contaminants from the environment when exposed to sunlight.

#### 3.6.2 Recycling of the ZnS-TGA photocatalysts

**Fig. 13:**
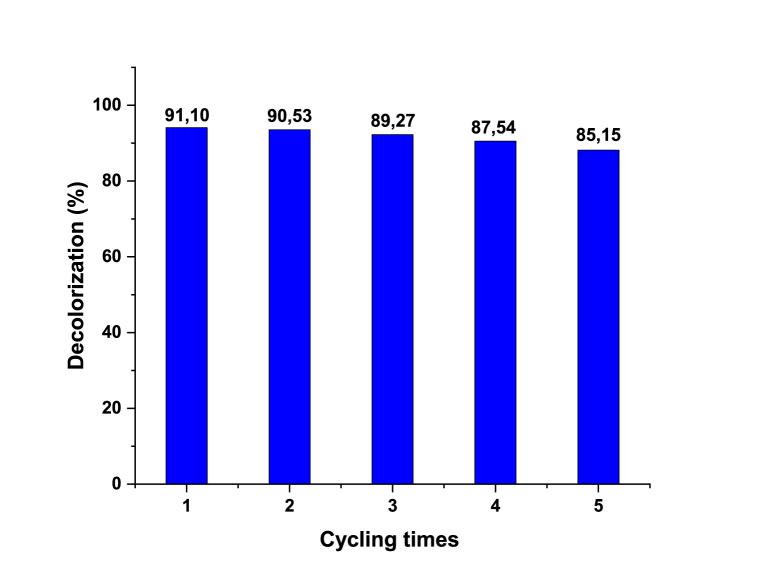
Results of the reusability of the ZnS-TGA NPs for the MB photodegradation.

The recycling of ZnS photocatalysts involves their recovery and reuse after driven the photocatalytic reactions. Reusability studies are required to evaluate the NPs long-term viability and help to make decisions about their use in different applications. NPs photocatalytic stability for MB dye degradation was tested for 5 continuous cycles, see the results reported in Fig. 13. NPs were collected after each cycle, rinsed with ethanol, and deionized water, and dried for 24 h at 100 °C before the next degradation cycle. Results showed that the photocatalytic efficiency remained nearly unchanged, and the MB degradation was 88 ± 3 % after 5 successive degradation cycles. These findings proved the high photocatalytic and physiochemical stability of NPs.

## 4 Conclusions

The impact of thioglycolic acid as a stabilizing agent on the surface layer of ZnS nanoparticles via the colloidal synthesis method was studied and analyzed in this study. This synthesis route allowed to obtain a colloidal material with low energy consumption, easy implementation, low cost and excellent purity. Surface characterization results via FTIR confirmed the functionalization of nanoparticles surface by TGA molecules, while the formation of nanocrystals with almost cubic phases was confirmed via XRD analysis. An average size of 7.15 nm was calculated with a specific surface area of 36.82 m^2^/g for the synthetized NPs. The synthesized nanoparticles were applied to remove MB dye from aqueous solution under sunlight irradiation where the photocatalysis-adsorption synergy was analyzed at different operating conditions. The maximum adsorption capacity for MB dye removal was 30.92 mg/g at pH 7 and 298 K, and this process was spontaneous and exothermic. The calculated adsorption energies ranged from 25.92 to 23.31 kJ/mol, implying the existence of physical interactions between the synthetized nano-adsorbent surface and MB dye molecules. Thiol-capped ZnS NPs exhibited a remarkable sunlight-based photocatalytic activity for MB dye degradation with a conversion rate of 60 % in the first hour. The sunlight-based photocatalytic activity of these nanoparticles achieved 91.1 % dye degradation efficiency in 180 min. The lowest MB concentration (e.g., 10 mg/L) provided the best MB dye degradation constant K (i.e., 0.025 min^−1^) with a half-time value of 47.46 min. The photocatalytic efficiency remained nearly unchanged, and the MB degradation was 88 ± 3 % after 5 successive degradation cycles. TGA-capped ZnS NPs can be considered as a promising photocatalyst prepared with non-toxic feedstock that can operate under sunlight irradiation for the environment detoxifying from organic pollutants.

## Statements & declarations

### Funding

Not applicable.

## Ethical approval

The manuscript describes original work and is not under consideration by any other journal. All authors approved the manuscript and this submission.

**Competing Interests:** The authors declared that they have no confict of interest.

**Consent to Participate:** All authors whose names appear on the submission made substantial contributions to the conception or design of the work; or the acquisition, analysis, or interpretation of results.

**Consent for publication:** All authors consent to this publication.

## Data availability

Data will be made available on request.

## CRediT authorship contribution statement

**Sabri Ouni:** Writing – original draft. **Faiza Yahia:** Writing – review & editing, Data curation. **Naim BelHaj Mohamed:** Writing – review & editing, Validation, Supervision. **Mohamed Bouzidi:** Writing – review & editing, Resources, Formal analysis, Data curation. **Abdullah S. Alshammari:** Writing – review & editing, Visualization, Validation, Supervision. **Fahad Abdulaziz:** Writing – review & editing. **Adrián Bonilla-Petriciolet:** Writing – review & editing, Validation. **Mansour Mohamed:** Writing – review & editing, Validation, Supervision, Conceptualization. **Ziaul R. Khan:** Writing – review & editing, Data curation. **Noureddine Chaaben:** Visualization, Supervision, Resources. **Mohamed Haouari:** Writing – review & editing, Validation, Supervision.

## Declaration of competing interest

The authors declare that they have no known competing financial interests or personal relationships that could have appeared to influence the work reported in this paper.
